# Brain Source Correlates of Speech Perception and Reading Processes in Children With and Without Reading Difficulties

**DOI:** 10.3389/fnins.2022.921977

**Published:** 2022-07-19

**Authors:** Najla Azaiez, Otto Loberg, Jarmo A. Hämäläinen, Paavo H. T. Leppänen

**Affiliations:** ^1^Department of Psychology, Faculty of Education and Psychology, University of Jyväskylä, Jyväskylä, Finland; ^2^Department of Psychology, Faculty of Science and Technology, Bournemouth University, Bournemouth, United Kingdom; ^3^Department of Psychology, Jyväskylä Center for Interdisciplinary Brain Research, University of Jyväskylä, Jyväskylä, Finland

**Keywords:** reading, ERPs, FRPs, auditory P1, auditory N250, visual N170, source reconstruction, brain correlates

## Abstract

Neural correlates in reading and speech processing have been addressed extensively in the literature. While reading skills and speech perception have been shown to be associated with each other, their relationship remains debatable. In this study, we investigated reading skills, speech perception, reading, and their correlates with brain source activity in auditory and visual modalities. We used high-density event-related potentials (ERPs), fixation-related potentials (FRPs), and the source reconstruction method. The analysis was conducted on 12–13-year-old schoolchildren who had different reading levels. Brain ERP source indices were computed from frequently repeated Finnish speech stimuli presented in an auditory oddball paradigm. Brain FRP source indices were also computed for words within sentences presented in a reading task. The results showed significant correlations between speech ERP sources and reading scores at the P100 (P1) time range in the left hemisphere and the N250 time range in both hemispheres, and a weaker correlation for visual word processing N170 FRP source(s) in the posterior occipital areas, in the vicinity of the visual word form areas (VWFA). Furthermore, significant brain-to-brain correlations were found between the two modalities, where the speech brain sources of the P1 and N250 responses correlated with the reading N170 response. The results suggest that speech processes are linked to reading fluency and that brain activations to speech are linked to visual brain processes of reading. These results indicate that a relationship between language and reading systems is present even after several years of exposure to print.

## Introduction

Learning to read is a complex multi-step process that requires both visual and auditory processes (Kavale and Forness, 2000; Norton et al., 2015; Vernon, 2016; LaBerge and Samuels, 2017). The question of whether speech processing and visual processing deficits are linked to reading disorders has been extensively addressed in the literature (Breznitz and Meyler, 2003; Breznitz, 2006; Wright and Conlon, 2009; Georgiou et al., 2012; Kronschnabel et al., 2014; Francisco et al., 2017; Karipidis et al., 2017; Ye et al., 2017). However, the nature of the link between the two modalities remains unclear (Gibson et al., 2006; Wright and Conlon, 2009; Blau et al., 2010; Georgiou et al., 2012; Ye et al., 2017; Rüsseler et al., 2018; Stein, 2018). Several studies have investigated this relationship using simultaneous auditory and visual stimuli in dyslexic vs. typical readers using behavioral and brain measures (Aravena et al., 2018; Karipidis et al., 2018; Fraga-González et al., 2021). In the present study, we investigated the extent to which speech processing at the brain level is associated with reading fluency and brain activity during reading. We examined these associations in a group of children with different levels of reading skills, ranging from poor to good.

Reading difficulty (RD), or dyslexia, is a frequent neurodevelopmental impairment that is commonly reported among school-age children. It involves a failure to acquire a satisfactory level of reading and spelling performance, despite normal intelligence and typical linguistic performance, in the absence of any organic, psychiatric, or neurological disorders, and despite adequate pedagogical opportunities (Démonet et al., 2004; Peterson and Pennington, 2015; Snowling et al., 2020). Dyslexia has been commonly linked to deficits in speech processing (Schulte-Körne et al., 1998; Kujala et al., 2000; Bishop, 2007; Abrams et al., 2009; Hämäläinen et al., 2013; Christmann et al., 2015; Lizarazu et al., 2015; Gu and Bi, 2020) and phonological processing (Snowling, 1998; Richardson et al., 2004; Vellutino et al., 2004; Christmann et al., 2015; Smith-Spark et al., 2017; Goswami, 2019).

A frequently reported problem in dyslexia is word decoding, which is mainly described as a deficit in reading speed, accuracy, or spelling difficulties (Snowling, 2001; Vellutino et al., 2004; Siegel, 2006; Hulme and Snowling, 2014). According to phonological theory, RD is caused by alterations in brain functions, such as a deficit in phonological representations, an information storing dysfunction, or information inaccessibility (Ramus and Szenkovits, 2008; Hoeft et al., 2011; Boets et al., 2013; Hornickel and Kraus, 2013; Prestes and Feitosa, 2017). Based on this theory, one of the main hypotheses underlying the mechanism of reading disability is the creation of phoneme-grapheme neural connections or inadequate representations when processing speech signals. This deficit could result from an alteration of the process of decoding grapheme-phoneme correspondences when decoding single letters, letter clusters, or words while reading (Goswami, 2000; Prestes and Feitosa, 2017). Weakness in building a stable network by binding letters and sounds eventually leads to reading problems (Goswami, 2002; Noordenbos et al., 2012; Vogel et al., 2013). Several studies of brain responses in children with reading difficulties have reported deficits in speech and phonological processing (Snowling, 1998; Castles and Friedmann, 2014; Ramus, 2014; Catts et al., 2017), with atypical phonological or phonetic representations of speech sounds shown to alter normal phoneme and word identification. Alternatively, an impairment in letter-speech sound mapping has also been suggested to be the origin of reading problems (Ehri, 2005; Maurer et al., 2010; Žarić et al., 2014; Fraga-González et al., 2015). Several studies have shown that speech processing is tightly linked to reading processes and reading skills (Pennington and Bishop, 2009; Zhang and McBride-Chang, 2010; Price, 2012; Duncan, 2018). The early ERP response, P1/N1-P2/N2 complex, is known to reflect basic phonological processes such as sound detection and identification and complexity processing (Maurer et al., 2002; Alain and Tremblay, 2007; Durante et al., 2014; Hämäläinen et al., 2015). Another response, the N2/N250, which is also part of the early complex, has been described in the context of syllable processing and interpreted to reflect the building of neural representation with repeated auditory stimuli (Karhu et al., 1997; Ceponiene et al., 2005; Vidal et al., 2005; Hommet et al., 2009; Hämäläinen et al., 2018; Wass et al., 2019). Studies have shown that basic speech processing was a strong predictor of infants' and young children's reading skills development as early as 6 months of age (Leppänen et al., 2002; Meng et al., 2005; Boets et al., 2011; Hayiou-Thomas et al., 2017; Lohvansuu et al., 2018).

Using the electroencephalography (EEG) technique, letter-sound mapping was investigated in typical (CTR) and dyslexic readers, and the quality of letter-speech sound processing was shown to be related to reading fluency, with evidence of a relationship between the auditory and visual modalities (González et al., 2016; Moll et al., 2016; Karipidis et al., 2018). This grapheme-phoneme bind created during cross modalities network coactivation, has been described as a key step for developing fluent reading (Chyl et al., 2018; He et al., 2021) by enhancing the specialized visual areas related to print when presented with the corresponding letter-speech sound. This process typically occurs in the early learning stages of reading (Ehri, 2005; Fraga-González et al., 2021). As an example of this effect in EEG studies, it has been shown that ERP amplitudes (for the mismatch responses MMN and LDN, for example) were enhanced when speech sounds were presented to typical readers with letters—an effect that was absent in dyslexic readers (Froyen et al., 2009)—suggesting that in atypical reading development, this letter-speech bind is absent or very weak. Similar results were reported in adults. Blau et al. (2009) investigated whether phonological deficits impaired the mapping of speech sounds into equivalent letters. The authors showed reduced audiovisual integration among dyslexics compared to controls, which was linked to reduced activation of the superior temporal cortex, reflecting a deficit in auditory speech processing. The importance of the auditory cortex in the integration of letter-speech sounds has also been addressed in functional magnetic resonance imaging (fMRI) studies, both in adults (Van Atteveldt et al., 2004; Holloway et al., 2015; Yang et al., 2020) and in children. Yang et al. (2020) studied the neural basis of audiovisual integration deficits in dyslexic children *via* fMRI. Based on brain activation analysis, the authors reported a less developed correspondence of orthographic and phonological information matching in dyslexic children. They also reported reduced functional connectivity of important brain structures involved in integration processes, such as the left angular gyrus and the left lingual gyrus. This difference in the left superior temporal gyrus (STG) between the two groups of children was supported by previous findings in literature, whereas the angular gyrus (AG) activity was mainly related to task demand and attentional processes.

Visual processing deficits in reading have also been reported for dyslexia and reading problems (Eden et al., 1996; Lobier et al., 2012, 2014; Giofrè et al., 2019; Archer et al., 2020). Visual deficits related to reading have previously been reported at different levels, such as in the sensory, temporal, attentional, and memory processes (Farmer and Klein, 1995; Snowling, 2001; Facoetti et al., 2006; Boets et al., 2008; Wright and Conlon, 2009; Conlon et al., 2011; Goswami, 2015). For example, low-level visual processing in letter-speech sound integration was addressed using a mismatched paradigm to investigate the influence of speech sounds on letter processing. Despite previous evidence of the systematic modulation effect of speech sound processing on letter processing, the reverse effect was not found (Froyen et al., 2010). The emergence of letter-speech sound correspondence has been studied in children *via* different neuroimaging techniques. Brem et al. (2010) studied the establishment of a reading network *via* speech processing in beginning readers *via* ERP and fMRI. That study focused on the left occipitotemporal cortex underlying the VWFA. The authors showed that print sensitivity in this area emerged in the early phases of reading acquisition, highlighting the critical role of VWFA in sound-print mapping. The results of Brem et al.'s investigation of fMRI and EEG data clearly indicated brain activity enhancement in the occipitotemporal area after the establishment of speech-print mapping through training. The authors reported that the auditory network involved was not the only one, but that a visual network was clearly co-activated during the coding-decoding phases, which highlighted the importance of the VWFA in this learning process. Brem et al. also associated the activation of this brain area with the visual N1 response of the ERP component sensitive to print, more commonly named N170. Pleisch et al. (2019) studied differences in reading processes between typical and dyslexic first-grade children by measuring the neural activation of the N1 response to print *via* combined EEG–fMRI methods. A differential modulation reflecting sensitivity to print was found only in typical readers in the ventral occipitotemporal cortex. The authors concluded that functional brain alterations in the language network play a role in dysfluent reading development.

The role of speech and language as the basis for reading is well established, where most dyslexics show difficulties in phonological processing (Siegel, 2006; Navas et al., 2014; Giofrè et al., 2019). Sensory or orthographic visual processing deficits have only been observed in a subgroup of the dyslexics (Wright and Conlon, 2009; Giofrè et al., 2019). Visual processing in RD remains an important processing aspect to study in reading research, which has already been a focus of investigation in the literature (Salmelin et al., 1996; Lobier et al., 2014; Archer et al., 2020). However, the ties between visual and auditory information processes in the context of reading vs. speech processing remain unclear. The processing of several letters in a short timeframe is an important skill for developing fluent reading. It has been shown that RD is characterized by slow word recognition and a higher error rate compared to typical reading (Ozeri-Rotstain et al., 2020). Efficient word processing depends on parallel visual processing of multiple letters (Lobier et al., 2012), where a problem in letter pattern perception leads to a problem in orthographic processing, inducing reading problems (Georgiou et al., 2012).

Monzalvo et al. (2012) used fMRI to investigate cortical networks for vision and language by comparing cortical activity in minimally demanding visual tasks and speech-processing tasks. In the visual paradigm, objects, faces, words, and a checkboard were used as stimuli presented in different blocks, and short sentences in native and foreign (unfamiliar) languages were used in the speech processing paradigm. Both visual and spoken language systems have been reported to be impaired in dyslexics. Monzalvo et al. found that dyslexics had reduced activation of words in the VWFA in the visual task and reduced responses in different brain areas, including the posterior temporal cortex, left insula, planum temporal, and left basal language area, extending to the VWFA, in the speech tasks, and the authors concluded that there was hypoactivation in the VWFA for written words and speech listening. These results highlight the role of the VWFA as an associative area in the processing of both types of stimuli: visual information in reading and auditory information in speech processing. A more recent fMRI study by Malins et al. (2018) used a task of matching printed and spoken words to pictures and found a significant correlation between the neural activity of both print and speech and reading skills in children. The authors studied trial-by-trial neural activation of different brain areas and their relationship to reading. They showed that the variability of the neural activation to print was positively correlated with the activation variability of the inferior frontal gyrus providing an additional evidence on the relationship between reading skills and sound processing. The authors discussed the common neural activations for print and speech and highlighted individual differences.

When studying visual processing, the eye-tracking technique is frequently used to examine visual processes and eye movements to investigate reading and reading disorders (Jainta and Kapoula, 2011; Tiffin-Richards and Schroeder, 2015; Kim and Lombardino, 2016; Nilsson Benfatto et al., 2016; Jarodzka and Brand-Gruwel, 2017; Breadmore and Carroll, 2018; Robertson and Gallant, 2019; Christoforou et al., 2021). FRPs are a specific type of ERP that rely on eye fixations and their corresponding brain activity (Baccino, 2011). This combined technique is commonly used to investigate reading (Baccino, 2011; Wenzel et al., 2016; Loberg et al., 2019; Degno and Liversedge, 2020). The FRP is based on EEG measurements of brain activity in response to visual fixations obtained by extracting the signal-averaged time-locked to the onset of eye fixations (Baccino, 2011). Fixations in reading are known to reflect the online cognitive process of several factors, such as the duration and location of a word, word frequency, and predictability. This process occurs in a series of events, starting with the transmission of the visual signal of the printed word from the retina to the visual cortex, visual encoding, initiation of word identification, and programming the next eye movement (Degno and Liversedge, 2020). A commonly used measure for studying individual differences in reading is first-pass fixation duration. This measure reflects the cognitive components of early visual processing, word identification, attention shifts, and oculomotor control (Zhang et al., 2021a). Jainta and Kapoula's (2011) study of eye fixations in reading showed a large fixation disparity that caused unstable fixations in dyslexic children when reading sentences. The authors concluded that visual perturbation may cause letter/word recognition and processing difficulties that lead to reading disorders. Zhang et al. (2021a) used first-pass fixation in sentence reading to investigate the brain network in natural reading. They showed that seed regions in the early visual cortex, VWFA, and eye-movement control network were associated with individual reading performance and brain connectivity in a resting state.

Interestingly, this visual dysfunction was not found systematically, since some studies did not report any differences between RD and typical readers and not all children with RD show a visual deficit.

In the context of RD, both speech and visual processes have only rarely been investigated *via* the ERP method. For example, Bonte and Blomert (2004a) investigated dyslexic readers' phonological processing in spoken word recognition using a priming paradigm. The authors examined the general ERP response and reading skills of beginning readers and reported deficits in N1 and N2 speech processes in dyslexics compared to controls. They interpreted these results as a phonological processing deficit reflecting the recruitment of different neural sources (Bonte and Blomert, 2004a). The N250 response, which is known to be part of the obligatory response (P1-N250), was also investigated in dyslexia, and previous studies showed a different response in this component in the RD group compared to the control group (Lachmann et al., 2005; Lohvansuu et al., 2014). The N250 is thought to represent low-level auditory processing, such as sound detection or feature extraction, but it is also part of a critical processing stage, which is the formation of the neuronal representation of sound/speech stimuli (Karhu et al., 1997; Hämäläinen et al., 2015). As reading involves the ability to convert print into sound, it is critical to further investigate the N250 response and its relationship to reading, as previous evidence has shown differences in this component between good readers and dyslexics. However, the relationship between N250 and reading remains unclear. In addition to the N1-N2 findings, later ERP responses were also found deficient among RD participants, such as the P3a, the N400, and the LDN (Hämäläinen et al., 2008, 2013; Jednoróg et al., 2010; Desroches et al., 2013; Leppänen et al., 2019). These findings provide evidence that speech processing may be altered in dyslexics at different stages of processing and at different latencies.

The brain potential of interest in reading is the N170, an ERP component that peaks between 150 and 200 ms, with a peak around 170 ms and a temporo-occipital negative topography (Rossion et al., 2002; Maurer et al., 2005b; Sánchez-Vincitore et al., 2018). The N170 has been identified as reflecting facial recognition and being sensitive to facial expressions (Blau et al., 2009; Hinojosa et al., 2015; Wang et al., 2019). This component is known to be sensitive to orthographic processing (Rossion et al., 2003) and to letters strings/words in reading. When left lateralized, the N170 has been shown to be a reliable physiological marker of reading and reading skills (Maurer et al., 2005b, 2008; Lin et al., 2011; Hasko et al., 2013; Zhao et al., 2014; Lochy et al., 2016). For example, it was studied in dyslexic children compared to controls, where the N170 was shown to have a larger response in the dyslexic group compared to controls (Fraga González et al., 2014; González et al., 2016). Time-locked to the visual response, this ERP response becomes a strong indicator for studying the dynamics of the visual cognitive processes (labeled FRP N170) of reading and reading disorders (Dimigen et al., 2011, 2012; Kornrumpf et al., 2016; Loberg et al., 2019; Dimigen and Ehinger, 2021).

In the present study, we investigated how the basic speech ERP responses—the P1-N250—are related to reading process, and how the visual FRP response in reading—the N170, which is known as a reliable marker of reading processes (Maurer et al., 2005b; Hasko et al., 2013)—are associated with reading skills in the same children. Previous evidence has shown a link between speech perception and reading, with speech processing being a predictor of reading development at an early age, but the temporal-brain dynamics remain unclear. Moreover, the question of whether this relationship remains present after the development of reading skills has scarcely been investigated. Here, we aim to investigate whether the basic processes of speech remain associated with basic processing of reading in school-aged children who have established a reading network, and how their reading skills may reflect their neuronal activity. This study represents a new approach to investigate how visual reading and auditory speech processes may be interlinked and linked to reading skills by combining different methods (ERP, FRP, and CLARA) for high temporo-spatial analysis.

Both auditory and visual modalities were tested in two separate tasks: a speech perception task and a sentence-reading task. We used source reconstruction with correlation analyses to identify the link(s) among reading skills and auditory processes, reading skills and visual processes, and the neuronal activity of the two modalities. This enabled us to study the brain dynamics of these processes by examining the neuronal origin of brain activity at the source level and to explore its relationship to reading skills. Based on previous evidence, we hypothesized that speech perception basic responses (P1-N250) would show correlations with reading skills (Bonte and Blomert, 2004a; Lohvansuu et al., 2018) and that the visual N170 response would also correlate with reading skills (Maurer et al., 2008; Mahé et al., 2013; Fraga González et al., 2014). Furthermore, we expect to observe a relationship between the speech processes P1 and N250, and the visual reading processes over the VWFA within the same subjects in these two independent tasks.

## Materials and Methods

### Participants

A total of 440 children from eight schools in the area of Jyväskylä, Finland, participated in three test cohorts. The study included a subsample of 112 children, all Finnish native speakers aged between 11 and 13. These children were invited to participate in the eSeek project (Internet and Learning Difficulties: A Multidisciplinary Approach for Understanding Reading in New Media). The participants were grouped based on their reading fluency scores derived from three different reading tasks. The latent score was computed for reading fluency using principal factor analysis (PAF) with PROMAX rotation in the IBM SPSS 24 statistical program (IBM Inc.). This score was based on the following three tests: The Word Identification Test, a subtest of standardized Finnish reading test ALLU (Lindeman, 1998) (factor loading 0.683); the Word Chain Test (Nevala and Lyytinen, 2000) (factor loading 0.683); and the Oral Pseudoword Text reading (Eklund et al., 2015) (factor loading 0.653).

The word identification test included 80 items, each consisting of a picture and four alternative written words. The task was to identify and connect correct picture–word pairs. The score was the number of correctly connected pairs within the 2 min. The word chain test consisted of 25 chains of four words written without spaces between them. The task was to draw a line at the word boundaries. The score was the number of correctly separated words within the 90 s time limit. The oral pseudoword text-reading test consisted of 38 pseudowords (277 letters). These pseudowords were presented in the form of a short passage, which children were instructed to read aloud as quickly and accurately as possible. The score was the number of correctly read pseudowords divided by the time, in seconds, spent on reading (for details, Kanniainen et al., 2019).

This reading score was computed for the whole sample for each subject. Children who scored below the 10th percentile were identified as poor readers (RD) and those who scored above the 10th percentile were identified as good readers (CTR).

All participants scoring equal to or below 15 points (10th percentile) in the cognitive non-verbal assessment testing were excluded. This assessment included a 30-item version of Raven's progressive matrices test (Raven and Court, 1998). Attentional problems were screened *via* the ATTention and EXecutive function rating teacher inventory (ATTEX in English and KESKY in Finnish) (Klenberg et al., 2010). To be included in the analyses, the participants had to score below 30 points on this test. Children with clear attentional problems were excluded from the study.

The brain response analyses were conducted on 112 participants: auditory data: 86 CTR participants (43 females and 43 males; age range = 11.78–12.84 years; mean age 12.36 years, SD: 0.27) and 26 RD participants (eight females and 18 males; age range = 11.84–12.94; mean age 12.31 years, SD: 0.34). Preprocessing and source modeling were performed on 92 participants' reading data: 65 CTR participants and 27 RD participants.

The correlation analysis only included participants with valid auditory and visual data. Sixty of these participants comprised the final CTR group (30 females and 30 males; age range = 11.88–12.84 years; mean age 12.37 years, SD: 0.28) and 20 participants were in the RD group (six females and 14 males; age range = 11.84–12.94 years; mean age 12.34 years, SD: 0.36). The final group, which included both samples from CTR and RD (labeled CTRD), comprised 80 subjects and was tested for normality and skewness. The tests showed a normal distribution and no skewness. For details, see the Supplementary Material.

None of the participants declared any auditory problems, and they all had normal or corrected vision with no history of neurological problems or head injuries. The current study was conducted in compliance with the Declaration of Helsinki, and the study protocols were approved by the Ethics Committee of the University of Jyväskylä, Finland. All of the methods used were performed in accordance with university guidelines and regulations. The participants and their parents provided signed informed consent prior to the study.

### Materials and Procedures

#### Auditory Materials and Stimulus Presentation

The auditory stimulus used for this study was originally presented in a passive oddball paradigm designed for another study, comprising a standard stimulus and two deviant stimuli presented over a duration of 10 min. The target stimulus (standard) was presented 800 times in the paradigm, but only 200 trials, which were the pre-deviant standard stimulus responses, were used for the analysis. These trials are believed to have the strongest representations of stimuli due to repetition. The stimulus consisted of a Finnish monosyllabic word *suu* (which means “mouth” in English), a basic, frequent, short, and easy word that is commonly used by itself in the Finnish language but could also be part of other words like [o**suu**s (“a portion or contribution”) or a**suu** (“lives”)]. This is also one of the first words learned by Finnish children at a very early age and is therefore expected to have a strong neural representation among Finnish participants. The stimuli were recorded by a male native speaker and were pronounced in a neutral manner. The recording was equalized and normalized in segmental durations, pitch contours, and amplitude envelopes using Praat software (Boersma and Weenink, 2010) for a more detailed description of stimulus preparation (Ylinen et al., 2019). The stimuli were presented *via* a loudspeaker placed on the ceiling ~100 cm above the participants' ear position and were presented at ~65 dB. The stimulus volume level was calibrated before each recording with a sound level meter (Brüel and Kjaer) placed on a pedestal device at the participant's head position (with the following settings: sound incidence = frontal; time weighting = fast; ext filter = out; frequency weighting = A, range = 40–110 dB; display = max).

#### Reading Materials

Two hundred sentences, each with between five and nine words, and a median length of six words, were used as visual stimuli. The sentences were presented in 20-point Times New Roman font on the screen in a free-reading task. Each letter was subtended at an average visual angle of 0.4 degrees on the screen, where the distance of the participants was ~60 cm from the monitor. A total of 912 words, with lengths varying from 5 to 13 letters, were included in the FRP analysis. The materials for this paradigm were part of a previous study. For a detailed description, see Loberg et al. (2019).

### Data Measurements

EEG recordings were performed in a sound-attenuated and electrically shielded EEG laboratory room located at the University of Jyväskylä facilities. There was no task for the auditory paradigm. Each child was instructed to minimize movement while listening passively to auditory stimuli. To maintain the child's interest in the experiment, he/she watched a muted cartoon movie playing on a computer screen. In the reading paradigm, the measurement was performed in the same room using a dim light. The child was instructed to freely read different sentences that appeared on the screen. During the recordings, the experimenters observed the participant *via* live video camera streaming and monitoring from a separate control room to ensure the wellbeing of the participant and that the experiment proceeded as expected.

Both EEG datasets were recorded with 128 Ag-AgCl electrode nets (Electrical Geodesic, Inc.) with Cz as the online reference, using NeurOne software and a NeurOne amplifier (MegaElectronics Ltd., new designation Bittium). The data were sampled online at 1,000 Hz, high-pass filtered at 0.16 Hz, and low-pass filtered at 250 Hz during the recording. The experimenter aimed to keep impedances below 50 kΩ and the data quality was checked continuously. All necessary adjustments or corrections were performed during short breaks and between the experiments' blocks to maintain good quality throughout the measurements.

The Eyelink 1,000 with 2,000 Hz upgrade (SR research) version was used for the eye-movement data acquisition of the reading task using a 1,000 Hz sampling rate. The sentences were presented on a Dell Precision T5500 workstation with an Asus VG-236 monitor (1,920 × 1,080, 120 Hz, 52 × 29 cm). At the beginning and the end of each trial, the synchrony between the two measures was ensured with a mixture of transistor-to-transistor logic pulses (to EEG) and Ethernet messages [to eye tracking (ET)]. The participants held their heads in a chinrest during the measurements. The calibration routine consisted of a 13-point run of fixation dots performed before each block and before each trial. This reading task was divided into four blocks. If the fixation diverged from the calibration by more than one degree, the calibration was redone. The experiment's trial started only upon the experimenter's approval of the calibration. Once the task started, the participants were instructed to press a button to move to the next trial (for details, see Loberg et al., 2019). The participants were instructed to read as quickly as possible. The quality of the EEG and the ET was maintained throughout the experiment, and corrections and recalibrations were performed as required. Short breaks were taken when needed or upon the participant's request.

In both experiments, the participants were informed that they were allowed to terminate the experiment at any time in the case of discomfort.

### Auditory Data Preprocessing

BESA Research 6.0 and 6.1 were used for offline data processing. Bad channels were identified from the data (number of bad channels: mean: 5.6, range: 1–13). Independent component analysis (Infomax applied to a 60-s segment of the EEG) (Bell and Sejnowski, 1995) was used to correct the blinks from each subject's data. Epoch length was set from −100 ms (pre-stimulus baseline) to 850 ms. The artifact detection criterion was set to a maximum of 175 μV for amplitude fluctuations within the total duration of the epoch. A high-pass filter of 0.5 Hz was set before averaging. Bad channels showing noisy data were interpolated using the spherical spline interpolation method (Ferree, 2006). The data were re-referenced offline to average the reference and averaged individually and separately for the standard stimulus.

### Reading Data Preprocessing

The co-registered EEG-ET data were processed in MATLAB using EEGLAB (v14.1.2) with an EYE-EEG (0.85) add-on. A high-pass filter at 0.5 Hz and a low-pass filter at 30 Hz were applied. Synchronization between the raw gaze position data and the EEG data was performed using shared messages in both data streams at the beginning and the end of each trial. Gaze positions outside the screen were automatically discarded. Discarded trials included all zero gaze positions resulting from blinks and between trial gaps in the recordings. All fixations corresponding to all the words within the sentences, except for the last word, during a first-pass reading were used to compute the FRP estimate. The responses were locked to the first fixation of each word, mean word length of 8, and saccade amplitude of 1,8798'. A time window of 100 ms was also considered bad data before and after these values. A binocular median velocity algorithm for detecting fixations (and saccades) was applied to the remaining gaze positions.

### Deconvolution Modeling of FRPs

The UNFOLD toolbox (Ehinger and Dimigen, 2019) was used for the FRPs estimation. The FRPs were estimated *via* a generalized linear model that was used for response estimation and the correction of overlaps between the responses with a generalized additive model for non-linear predictors (Loberg et al., 2019). The modeled response ranged from −700 to 500 ms from fixation onset. All blink time points, eye movements outside the screen, and segments with large fluctuations were removed from the response estimates. Fixations on the target word during re-readings were excluded from the FRP estimation.

### Source Reconstruction and Spatial Filtering

**Figure 1:**
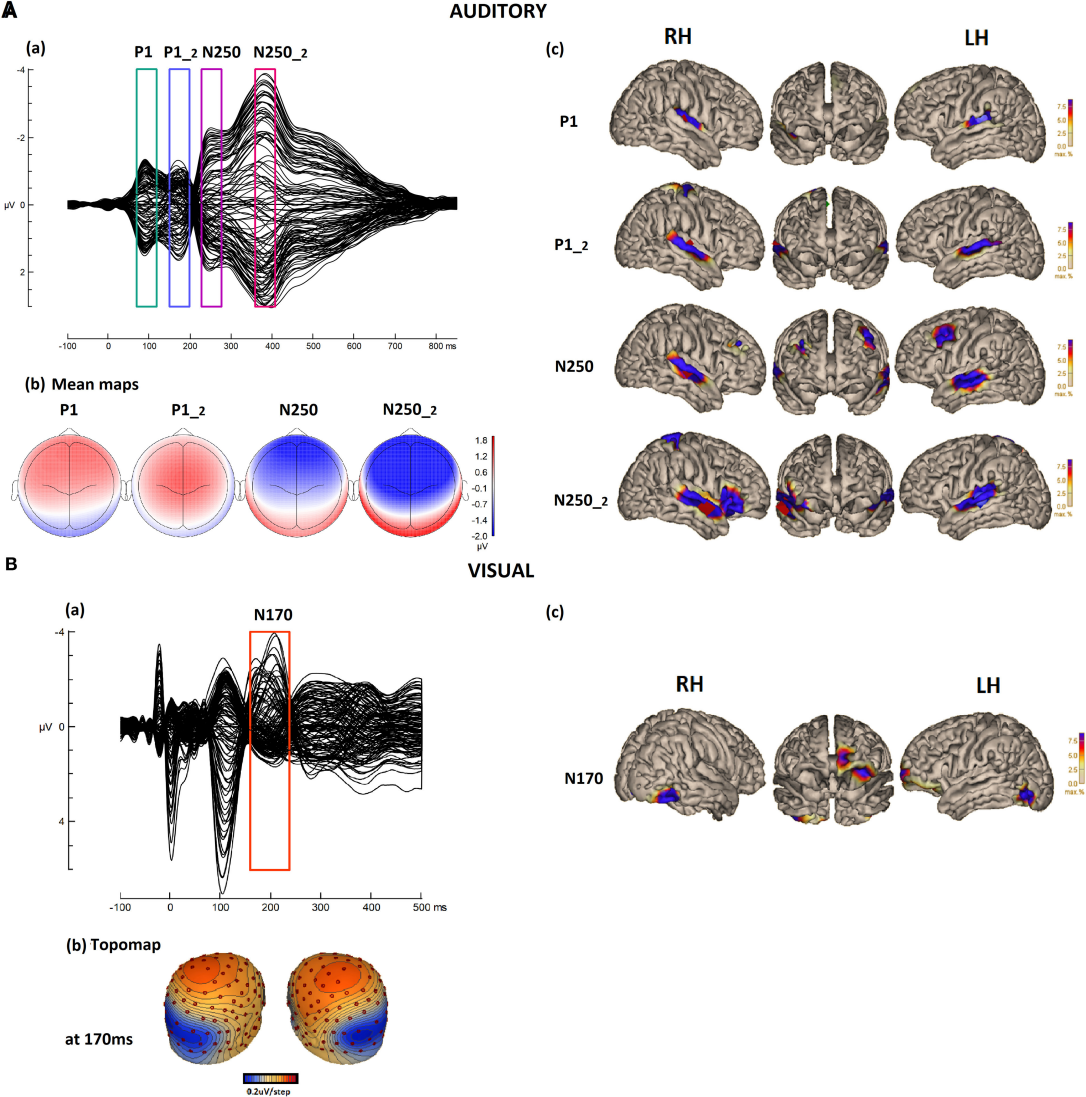
**(A)** (a) Auditory/speech ERPs in the CTRD group (*N* = 80) grand average. Butterfly plots for the responses to the standard stimulus “suu” over 129 electrodes. The boxes around the peaks indicate P1, P1-2, N250, and N250-2 responses. (b) The corresponding mean topographic maps for the time windows of 70–120 ms (P1), 150–200 ms (P1-2), 230–280 ms (N250), and 360–410 ms (N250-2), respectively. (c) Cortical CLARA reconstruction for each component. **(B)** (a) Visual/reading FRPs in the CTRD group (*N* = 80) grand average. Butterfly plots for the responses to word stimuli over 129 electrodes. (b) The topographic map of N170 at 170 ms and (c) its cortical source CLARA reconstruction.

Source analyses were conducted using BESA Research 6.1 and 7.0 to estimate the active sources in the speech processing and reading tasks. The neuronal sources were estimated *via* an inverse approach with a distributed source model in the brain volume: classical LORETA analysis recursively applied (CLARA) restricted to the cortex. For accurate forward head modeling, an appropriate FEM head model for 12-year-olds was implemented. Model solutions were created based on the group ERP brain source reconstructions for each brain component for the CTRD group combined in a unique model. For the auditory data, source locations were calculated for P1, P1-2, N250, and N250-2 (see an illustration of the ERP auditory responses in Figure 1). Model solutions were similarly computed for the reading data based on the group FRP estimates, where the target component was N170. The source analysis was performed ~10 ms before the peak for all components. This time point was chosen after inspection and after searching for the best solutions for the different responses. This time showed the best modeling solution for the source activity, with the clearest sources and the best residual variance. These group-based solutions were used to create a standard model to filter cortical sources, and only sources that were found to be activated in the common group (CTRD) were included in the final model. For each CLARA source identified, a regional dipole was fixed to combine the power sum of the three orthogonal orientations of the regional sources. The regional sources were computed for each component. They were then used as spatial source filters and applied to individual data. The source filter generated individual solution waveforms for each participant. A mean scalar value for each subject was computed as the sum of the source activity measures at all time points over a time window between ~20 and 30 ms around the peak, specified for each component (a detailed description of the time windows is provided below).

### Correlations

Correlations between source activations were converted into scalar values for each modality, and the reading scores (PAF) were examined across the CTRD group using Pearson's correlation coefficients. For each source activity, the mean value was calculated around the peak using MATLAB R2019b (Mathworks^®^), as described above. For the auditory data, the time windows for the averages were 80–110 ms for P1, 150–180 ms for P1-2, 230–250 ms for N250, and 360–390 ms for N250-2. For the visual data, the time window 180–210 ms was used for N170. These time windows were chosen based on visual inspection of the group ERP and FRP grand averages. The time windows were fixed so that the peak was always located in the middle of the window.

Pearson's correlation coefficients were calculated between the average source activity and the reading score of the participants using IBM SPSS statistics 26 (IBM corp), version 26.0.0.1, and applying a false discovery rates (FDR) correction of q = 0.05 (Benjamini and Hochberg, 1995) for the brain-to-behavior correlations and the brain-to-brain correlations. Correlations within brain activity between auditory and visual source activities were computed. A partial correlation (controlling for reading skills/PAF) between the source activity in the reading and speech processes was also performed.

## Results

### Brain Responses and Source Reconstructions

#### Brain Responses to Auditory ERP and Visual FRP Data

The auditory grand average ERP and the different auditory components are illustrated in Figure 1A. The ERP waveform (Figure 1Aa) shows four components that emerged in response to the auditory stimulus. The first component peaked at around 90 ms, with a clear fronto-central positive polarity, and reflected the P1 response to the stimulus onset. This was followed by a second positive component peaking at around 170 ms, reflecting a second P1 response (P1-2) in response to the onset of the vowel or to the consonant-vowel transition. This response had a somewhat more central topography. The third component peaked at around 250 ms and reflected the N250 response to the stimulus onset, followed by a fourth component peaking at around 370 ms, most likely reflecting a second 250 (N250-2) response to the consonant-vowel transition or the onset of the vowel in the stimulus. Both responses showed clear negativity in the fronto-central area, with a larger amplitude for the second N250 response (Figure 1Ab).

The grand average of the FRPs during reading is illustrated in Figure 1B. The component peaking around 200 ms reflects the visual N170 response, with topography (Figure 1Bb) showing a typical N170 response. The polarity was positive over the central area and negativity in the occipital areas, with a preponderance toward the left occipital hemisphere.

### Cortical Sources in Speech Processing

The group-based cortical source reconstruction (applying CLARA) of the auditory responses is illustrated in Figure 1Ac. For auditory P1, the source reconstruction at 80 ms, shows a bilateral focal activation of the primary auditory cortices (A1) [with a total residual variance (RV) of 1.78%]. The source reconstruction of the second component P1-2 performed at 160 ms shows the activation of similar bilateral sources over the auditory cortices. This second response shows slightly larger activity covering a larger area than the first P1, with an additional small activation over the central region (total RV = 5.12%). The third source reconstruction performed at 230 ms for the first N250 response revealed four sources. Two sources were active bilaterally in the left and right temporal lobes at the level of the superior temporal area (STA). In addition, the inferior frontal area (IFA) in the left hemisphere and the middle frontal area in the right hemisphere were activated (total RV = 2.83%). The fourth reconstruction was performed for the N250-2 response at 370 ms. The source reconstruction showed four sources: bilateral activation of the left and right STA, the third source in the right IFA, and the fourth in the center-right area of the cortex (total RV = 2.19%). Only the bilateral auditory sources across the different components were used to run the correlation analysis to investigate the relationship between the auditory speech perception processes and the reading processes at both the behavioral and neuronal levels. The other sources were discarded because they are believed to reflect additional processes that are related to attentional or semantic processes.

### Cortical Sources in Reading Processing

The group-based cortical source reconstruction of the visual response is illustrated in Figure 1Bc. For reading N170, the reconstruction was performed at 190 ms and showed five main sources (with an RV of 6.07%). Two sources were located in the left and right occipital areas: one over the middle temporal area and one over the right visual cortex. Two additional activations were also found over the left frontal area: one source located in the left orbitofrontal area and the second in the left prefrontal area. Only the visual reading sources of the occipital areas were kept for the correlation analysis to investigate the reading processes, as the frontal sources are believed to reflect other processes that are mainly related to attentional processes.

### Correlations

#### Cortical Source Correlations With Reading Scores

Table 1 presents the correlations between the scalar values of the cortical source activity in the speech paradigm and reading scores, and in the cortical source activity in the reading paradigm and reading scores.

**Table 1:** Brain-to-behavior correlation analysis between reading fluency and brain source activity in auditory and visual sources.

	**Components**									
	**Auditory P1**		**Auditory P1_**2		**Auditory N250**		**Auditory N250_**2		**Visual N170**	
**Sources**	**R AC**	**L AC**	**R STA**	**L STA**	**R STA**	**L STA**	**R STA**	**L STA**	**R VWFA**	**L VWFA**
Correlation	−0.141	**−0.337**	−0.034	−0.192	−0.204	−0.096	**−0.304**	**−0.273**	−0.210	**−0.224**
Significance	0.212	**0.002** ^**a**^	0.762	0.880	0.690	0.396	**0.006** ^**a**^	**0.014** ^**a**^	0.062	**0.046**

*AC, auditory cortex; STA, superior temporal area; VWFA, visual word form area; R, right hemisphere; L, left hemisphere*.

*The correlations significant before the FDR correlation are shown in bold*.

a *indicates that the correlations remained significant after the FDR multiple comparison corrections*.

A significant negative correlation was found between the P1 source activity of the left auditory cortex (A1) and the reading score (PAF). The correlation analysis with the right source activity did not reveal any significant results. Neither the right nor the left brain activity of the P1-2 or N250 sources correlated with PAF. At the time window of the N250-2 response, source activities in both the left and right temporal areas (STA) correlated negatively with PAF. The correlations indicated that the larger the response, the poorer the reading score. The correlations between the scalar values of the visual sources and the PAF are illustrated in Table 1. Only the left occipital source activity located over the left occipital area (L VWFA) correlated negatively with the PAF score. However, this correlation became non-significant after multiple comparison corrections.

#### Correlations Between Visual and Auditory Sources

**Figure 2:**
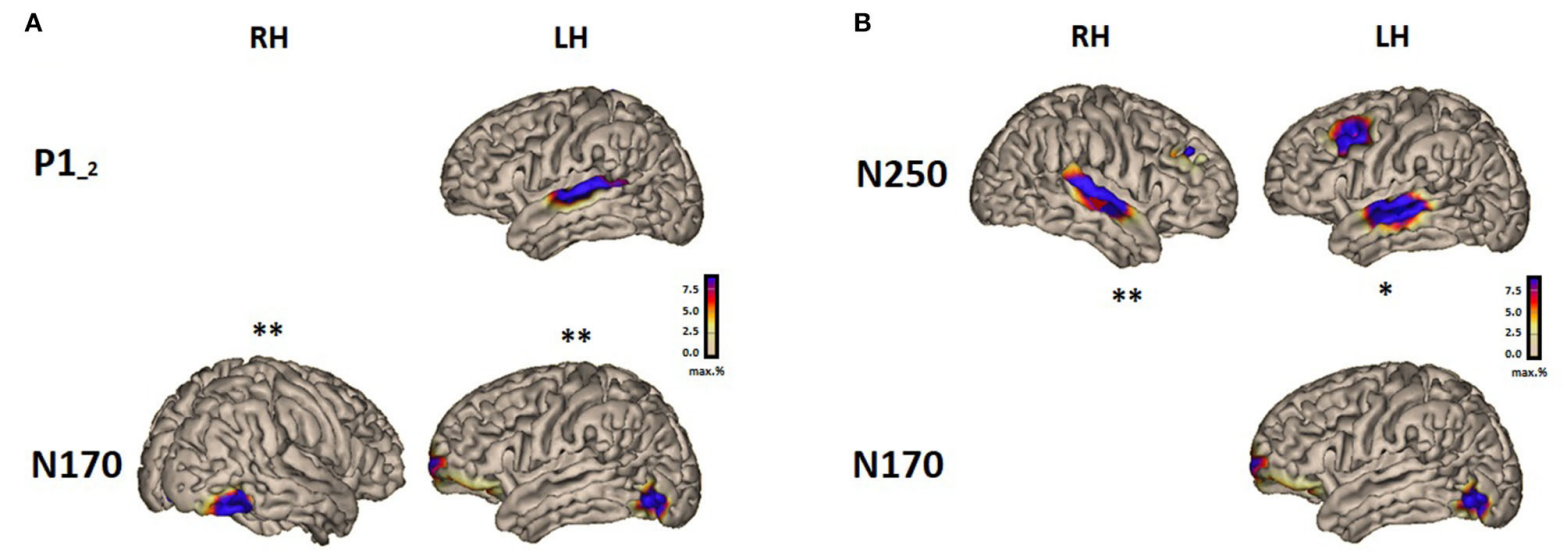
Summary results showing significant correlations between the source activity in speech processing and reading in the brain-to-brain analysis for the P1-2 vs. N170 **(A)** and N250 vs. N170 **(B)**.

Figure 2 shows the correlations between the scalar value of the visual N170 source and the auditory source activities.

The activity of the auditory P1-2 source (for consonant-vowel transition/vowel onset in “suu”) located in the left hemisphere over the temporal area (L STA) correlated significantly with both active sources of the N170 over the left and right hemispheres (L VWFA and R VWFA). The higher the auditory source activity, the higher the activity of the visual sources. The activity of the auditory N250 sources (for the stimulus “suu” onset) located in the left and right hemispheres (L STA and R STA) correlated significantly only with the left source activity of the N170 response (L VWFA). The larger the response to the auditory stimulus, the larger the response to the visual stimulus; see Table 2 for details.

**Table 2:** Brain-to-brain correlation analysis between auditory and visual source activity.

			**Auditory components**							
			**Auditory P1**		**Auditory P1_**2		**Auditory N250**		**Auditory N250_**2	
	**Sources**		**R AC**	**L AC**	**R STA**	**L STA**	**R STA**	**L STA**	**R STA**	**L STA**
Visual N170	L VWFA	Correlation	0.146	0.196	0.121	**0.335**	**0.294**	**0.286**	**0.222**	**0.231**
		Significance	0.197	0.081	0.284	**0.002** ^**a**^	**0.008** ^**a**^	**0.010** ^**a**^	**0.047**	**0.039**
	R VWFA	Correlation	−0.004	0.118	0.180	**0.316**	0.209	0.154	0.122	**0.225**
		Significance	0.972	0.299	0.109	**0.004** ^**a**^	0.063	0.172	0.279	**0.045**

*AC, auditory cortex; STA, superior temporal area; VWFA, visual word form area; R, right hemisphere; L, left hemisphere*.

*The correlations significant before the FDR correlation are shown in bold*.

a *indicates that the correlations remained significant after the FDR multiple comparison corrections*.

Partial correlations controlling for reading scores were conducted to investigate whether the brain-to-brain correlation was mainly driven by reading skill level. As shown in Table 3, controlling for the reading score did not change the correlations noticeably.

**Table 3:** Partial correlation (controlling for reading scores) between the auditory and visual source activities conducted for the brain-to-brain correlations (after FDR correction).

	**Components correlated**							
	**Auditory P1_**2 *Visual N170				**Auditory N250** *Visual N170			
**Sources correlated**	**Auditory** **L STA**	**Visual** **L VWFA**	**Auditory** **L STA**	**Visual** **R VWFA**	**Auditory** **R STA**	**Visual** **L VWFA**	**Auditory** **L STA**	**Visual** **L VWFA**
Correlation	0.306		0.288		0.260		0.273	
Significance	0.006		0.010		0.021		0.015	
df	77		77		77		77	

*STA, superior temporal area; VWFA, visual word form area; R, right hemisphere; L, left hemisphere; df, degree of freedom*.

## Discussion

This study had two main aims. The first was to investigate the relationship between speech processes and reading fluency, indicated by the PAF score, and visual brain activity in reading, as reflected by the VWFA activation, with the reading score. The second aim was to investigate the brain-to-brain responses for speech and reading processes among a group of children with different reading skills, ranging from good to poor. The study was conducted using brain ERPs for speech stimuli, FRPs for words in sentence stimuli, and source reconstruction for both processes to conduct the correlation analysis. To reveal the link between brain activity and reading skills, we first investigated the correlation between the brain activity of each modality (auditory and visual separately) and reading skills, as indicated by PAF, a reading fluency score derived from three different reading tasks. Our results showed that brain activity correlated with reading scores over the P1 and N250-2 components. The brain activity in reading, as reflected in N170 over the left hemisphere occipital area (L VWFA), correlated significantly with the reading fluency score. However, this correlation did not survive the statistical correction. The brain-to-brain analysis revealed the presence of significant correlations between speech-generated brain responses and reading source activity. The strength of the speech processing sources in the P1-2 and the early N250 showed a correlation with the VWFA source strength for N170. The current results are in line with the trends found in the literature, where the early speech components, P1 and N250, showed correlations with reading. However, our results showed that specific components correlate with behavioral reading skills, whereas other components correlate with brain reading processes. Our findings provide new evidence that there is still reliance on the auditory system and basic speech processes, even after long exposure to print, suggesting that the visual reading system continues to be linked to the auditory system at this developmental age.

In the first part of the study, we investigated the different brain components emerging in speech processing and reading tasks and their cortical sources. In the speech processing task, we examined brain responses to the standard “suu”. We chose this stimulus because it was the most repeated speech sound in the oddball paradigm. The literature has shown that stimulus repetition forms a strong memory trace (Jaramillo et al., 2000; Näätänen and Rinne, 2002; Haenschel et al., 2005) and generates a strong neural phonemic/phonetic representation. This phonetic representation was suggested to be linked to the print N170 response (Hsu et al., 2009; Zhang et al., 2021b).

The speech processing ERP results showed two main responses, P1 and N250, both of which have a two-peaked structure reflecting the nature of the syllable-word stimulus “suu”. Two similar positive peaks appeared in the early part of the response, one at 80 ms and the second at 170 ms, both of which showed similar scalp topographies with a fronto-central distributed positivity. The first peak seems to be a classic P1 peak emerging in response to the first sound of the syllable /s/, labeled here as P1. The second peak seems to emerge as a response to the second sound of the stimulus, /uu/, labeled as P1-2. This double-peak structure was also found for the second part of the response in the time range of the N250 component. Two similar peaks with similar fronto-central negative topographies appeared at 250 and 370 ms. The first N250 response is likely to reflect the further processing phase of the first sound /s/ (of /suu/), labeled as the early N250, and the second response to reflect the second processing phase of the second sound /uu/ and labeled as N250-2. N250 and N250-2 differed in amplitude, where the second component showed a very high negative amplitude compared to the first. This may be interpreted by a cumulative effect, where the N250-2 compromised the coarticulation processing in addition to the stimulus second sound /uu/ processing. This higher amplitude could also reflect the repetition effect, as both N250 and N250-2 showed higher amplitudes compared to the P1 responses. Another possible interpretation is that this enhanced response is due to the nature of the word stimulus, its strong familiarity, and its well-established neural representation. Early lexical/semantic access in this early phase is also possible. Early semantic access at this time range has been proposed in the literature (Zhao et al., 2016).

Previous studies have identified the early complex P1/N1-P2/N2 as the auditory change complex, reflecting the consonant-vowel transition in naturally produced syllables by children (Boothroyd, 2004). The P1-N250 complex response has been described in the literature as part of the basic auditory processing response (Ceponiene et al., 2005; Gansonre et al., 2018). The P1 is known to be an obligatory response reflecting sound detection and phoneme identification (Durante et al., 2014; Hämäläinen et al., 2015; Kuuluvainen et al., 2016), whereas the N250 was suggested to reflect phonological processing (Eddy et al., 2016), but also seemed to play a role in memory trace formation (Karhu et al., 1997; Ceponiene et al., 2005; Khan et al., 2011; Hämäläinen et al., 2013). These auditory speech responses have previously been shown to be linked to reading skills and have been studied in the context of typical reading and reading problems (Parviainen et al., 2011; Hämäläinen et al., 2015; Kuuluvainen et al., 2016). Differences between typical and dyslexic readers in these obligatory brain responses were found to emerge between 100 and 250 ms (Bonte and Blomert, 2004b; Hämäläinen et al., 2007, 2015; Khan et al., 2011).

In the reading task, the FRP results showed a typical N170 response. The N170 component has previously been described as reflecting objects and face recognition processes (Rossion et al., 2002; Collin et al., 2012; Hinojosa et al., 2015). It is also known to reflect print and word reading processes. This response was investigated in typical reading and RD and has been shown to have left-lateralized brain activity in reading (Maurer et al., 2005a, 2008; Mahé et al., 2013; Sacchi and Laszlo, 2016; Loberg et al., 2019).

In source reconstructions, the P1 component showed bilateral activation over the primary auditory cortices. In P1-2, the source reconstruction also shows bilateral brain activity in the auditory areas extending to the lateral surface of the STAs in this later response. The sources seem to be similar in both P1 responses, as both reflect similar processes occurring at different time points, where each component reflects the processing of a specific sound of the stimulus. Similar brain areas have been identified for P1 sources when processing auditory stimuli in adults and children (Godey et al., 2001; Shahin et al., 2004; Ruhnau et al., 2011). Our source reconstruction of the N250 component showed more inferior bilateral sources over the auditory areas (superior temporal and middle temporal areas), but an activation of frontal sources was also observed. In the N250-2, bilateral activation was also found in the auditory areas, with slightly more anterior location and with activation of frontal areas. Similar brain areas have previously been defined as the source origins of the N250 component to auditory stimuli (Parviainen et al., 2011; Hämäläinen et al., 2015) and speech processing (Ortiz-Mantilla et al., 2012). The STAs has been said to play a role in phonological (Hickok and Poeppel, 2007) and language processing (Trébuchon et al., 2013). The encoding of speech sounds in the STG was summarized in the review by Yi et al. (2019).

The source reconstruction of the P1-N250 complex showed the basic speech processing temporal and spatial dynamics of the stimulus, suggesting that these responses are more anteriorly located through time. Furthermore, our results suggest that the generators of the P1 and N250 components are different, although very closely located, with our source analysis suggesting more anterior and ventral sources for the N250 responses. The difference in source generators and topographies between the P1 and N250 responses clearly indicates two different processes. We argue that the P1 components seem to reflect the sound detection, phonetic processing, and feature extractions of each stimulus unit, whereas the N250 seems to reflect more complex processes, such as articulation processing and memory trace formation, as introduced above. The differences between the double peaks in P1 (P1 and P1-2) and N250 (N250 and N250-2) probably reflect the transitional state from one processing to the next, notably observed in the second components (P1-2 and N250-2) with slightly different auditory source locations in addition to the emergence of frontal sources. These frontal activations may reflect additional processes. These findings confirm our interpretations of the ERP responses.

The source reconstruction of the N170 shows bilateral activation of the occipital areas over the VWFA and activation of the left frontal area. The activation of VWFA as the source generator of N170 confirms previous findings. The N170 is known as the marker of visual specialization for print processing, and its relationship to the VWFA is well established in the literature (Maurer et al., 2005a; Maurer and McCandliss, 2007; Mahé et al., 2013). The left frontal activation is also in line with previous findings (Maurer et al., 2011). However, previous evidence showed a left lateralization of the N170/VWFA to be characteristic of the visual expertise of reading (Maurer et al., 2008). Interestingly, we observed bilateral activation over the occipital areas. N170 bilateral activation was previously reported in young children, indicating immature development of their reading systems (Uno et al., 2021). Our group sample of children comprise sixth-graders, who were exposed longer to print, but this group comprised both good and poor readers. Given that dyslexic readers have been shown to lack hemispheric lateralization of the N170/VWFA (Maurer et al., 2005a), the atypical activation observed in the right hemisphere in the source analysis most likely comes from the poor reader subsample. This atypical activation may also indicate an immature reading system in the RD subgroup.

The correlation analysis excluded frontal sources found in both speech ERP and reading FRP source reconstructions because they are known to be part of the attention network and the frontal eye field (Ptak, 2012).

In the reading process, N170 correlated with the reading scores, but it did not survive the statistical correction. The relationship between the N170 and reading was expected based on strong evidence in the literature showing the role of this visual component in reading and print processing (Maurer et al., 2005a; Hasko et al., 2013). In line with previous findings, correlation results between the N170 response and reading scores were found over the left occipital area. This left lateralization has also been described in the literature as the neural biomarker of the brain's sensitivity to print and word processing (Simon et al., 2007; Maurer et al., 2008; Zhao et al., 2012). However, it seems that the correlation we found was weak, as it did not survive the statistical corrections. One reason for this result is the methodological approach used in this study. As we have been computing FRPs for a group average containing 80 subjects and for multiple words, the effect may have been weakened through this averaging procedure.

The correlation analysis between cortical brain activity and reading scores in the auditory P1 response showed a significant correlation between left (primary) auditory cortex activity and reading score. Previous studies have shown that time cues and temporal acoustic information are typically processed by the left auditory cortex (Ladeira et al., 2011; Heimrath et al., 2016). Our results also suggest a left lateralization effect of the auditory P1 in response to speech stimuli, which is in line with previous findings. Interestingly, we found a negative correlation with reading skills, showing that the more active this brain area was, the lower the reading skills; this result contradicts previous findings (Shaywitz et al., 2002; Meyler et al., 2007). The smaller response observed in good readers may reflect the maturity of the neural network. Furthermore, correlations were not found in the right hemisphere for this component, which may suggest that brain activity in the right hemisphere may not be linked to reading skills.

N250-2 showed significant correlations between the reading scores and the STAs in both hemispheres. These brain areas were also shown to be part of the N250 component in typical auditory and language processing (Albrecht et al., 2000; Mody et al., 2008; Proverbio et al., 2011). This temporal activation was studied previously, and the role of the temporal areas was discussed in speech sound processing as reflecting low-level speech encoding (Hullett et al., 2016; Berezutskaya et al., 2017; Yi et al., 2019). The literature includes strong evidence of the role of the superior temporal area in reading and demonstrates the function of this brain area in relation to phonological processing in reading (Simos et al., 2000; Mesgarani et al., 2014).

All the correlations found between the auditory/speech brain activity and the reading scores or the visual/reading brain activity and the reading scores were negative. These results show that the more active the brain was, the lower the reading skills were. One possible interpretation is the recruitment of additional neuronal resources to compensate during atypical processing. Recruiting additional resources could be an adaptation to rebalance processing, as previously suggested in the literature (Lohvansuu et al., 2014). Another possible explanation for this result is the developmental phase of this age group. It has been suggested that visual reading skills follow an inverted U-shaped developmental trajectory (González et al., 2016). It is possible that in this age group, reading skills follow the inverted U-shaped curve of expertise in both the visual and auditory domains, which may explain the negative correlation.

We found correlations between brain activity to the visual stimuli and the auditory stimuli. The auditory source activity (in the STA) of the P1-2 response correlated significantly with both N170 sources in the left and right hemispheres (VWFA). The N250 sources (L STA and R STA) correlated only with the left N170 source (L VWFA). The N250-2 sources also showed correlations with the N170 sources over both hemispheres, but these correlations were weak and did not survive the statistical correction. Overall, these brain-to-brain correlation results suggest a strong relationship between the left occipital source in the reading processes and the auditory processes in both hemispheres. This result confirms our hypothesis, assuming that auditory and reading processes are interlinked and is grounded in the literature (Lin et al., 2011). Furthermore, the left lateralization found in the N250 correlation with the N170 is in line with the phonological mapping hypothesis. As this theory proposed that the left lateralization of the VWFA, the source origin of the N170 results from recruiting the left auditory language regions to link the orthography and phonology (Sacchi and Laszlo, 2016). Our correlation analysis suggests that the auditory region recruited for this purpose could be the STA as this area correlated with the VWFA. In addition, the positive correlation results suggest that both modalities behave in the same direction, so when brain activity is higher in one modality, it is also higher in the other modality. This may be interpreted by the presence of a compensatory or a complementary system that seems to act consistently across the two modalities.

Interestingly, the partial correlation analysis did not reveal a significant difference after controlling for reading. This result may indicate that the two modalities may be linked independently of the reading variable, suggesting the presence of possible common mechanism or network between the two modalities. This claim requires further investigation.

In line with our hypothesis, we found correlations between brain activity in speech processing and reading. Correlations between auditory and visual perception and reading have previously been shown on the behavioral level *via* meta-analysis (Kavale and Forness, 2000), and several studies have investigated both processes using simultaneous audiovisual stimuli. No such correlation was investigated *via* neuroimaging, as our findings showed the presence of correlation, even in independent tasks. With this method, we were able to investigate spatio-temporal processing in both processes and reveal, with high temporal accuracy, the different events, which allowed audiovisual sequential partial mapping in relation to reading. Our results confirmed earlier findings of auditory cortex responses to speech stimuli linked to reading skills, suggesting either the activation of the phonological route or the effect of learning to read through phonology still active at sixth grade when reading skills are fluent in most children. Similarly, the fusiform cortex or (STA) activity in response to print and correlation to reading skills confirms earlier findings and suggests this area is sensitive to environmental regularities, which seems to be linked to reading skills. From our results we were able to show the relation between the two routes, suggesting a link between the VWFA and STA.

## Data Availability Statement

The raw data supporting the conclusions of this article will be made available by the authors, upon reasonable request.

## Ethics Statement

The studies involving human participants were reviewed and approved by the Ethical Committee of the University of Jyväskylä. Written informed consent to participate in this study was provided by the participants' legal guardian/next of kin.

## Author Contributions

NA, OL, JH, and PL: conceptualization, writing, revising, and editing. OL: programming. NA: data collection, writing the main manuscript, and created the figures. NA and OL: data preprocessing and analysis. All authors contributed to the article and approved the submitted version.

## Funding

This work was supported by the Predictable (Marie Curie Innovative Training Networks, No. 641858), the Academy of Finland TULOS-program project eSeek-Internet and Learning Difficulties: A Multidisciplinary Approach for Understanding Reading in New Media (No. 274022), Niilö Helander foundation and the Department of Psychology at the University of Jyväskylä.

## Conflict of Interest

The authors declare that the research was conducted in the absence of any commercial or financial relationships that could be construed as a potential conflict of interest.

## Publisher's Note

All claims expressed in this article are solely those of the authors and do not necessarily represent those of their affiliated organizations, or those of the publisher, the editors and the reviewers. Any product that may be evaluated in this article, or claim that may be made by its manufacturer, is not guaranteed or endorsed by the publisher.
