# Supplementary material for: Brain Source Correlates of Speech Perception and Reading Processes in Children With and Without Reading Difficulties
Source: Front Neurosci. 2022 Jul 19;16:921977. doi: 10.3389/fnins.2022.921977 (PMC9344064; doi:10.3389/fnins.2022.921977)
Supplement: Supplementary file 1 [file Data_Sheet_1.docx]

Supplementary Material

# Supplementary figures

#
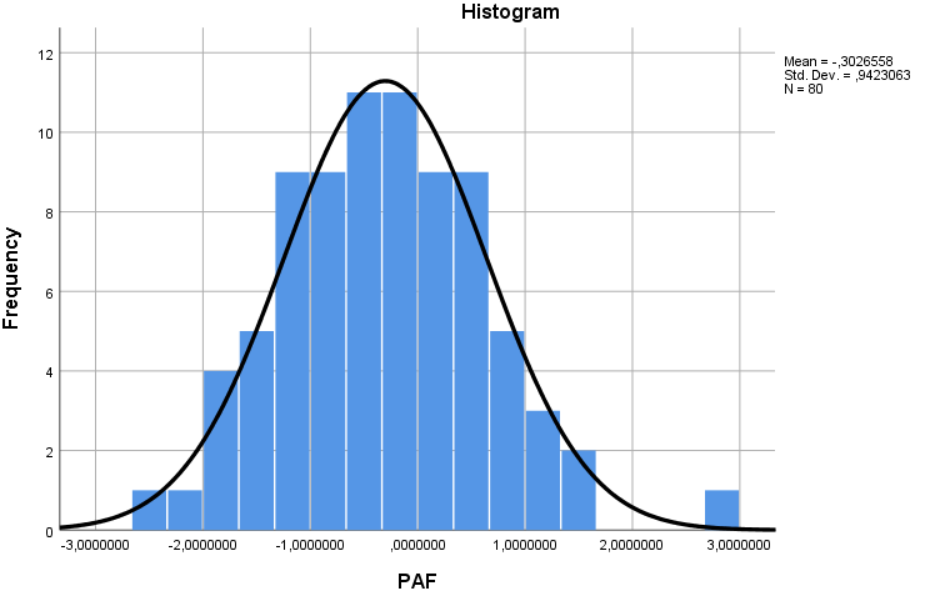


# Supplementary Figure 1. PAF normality


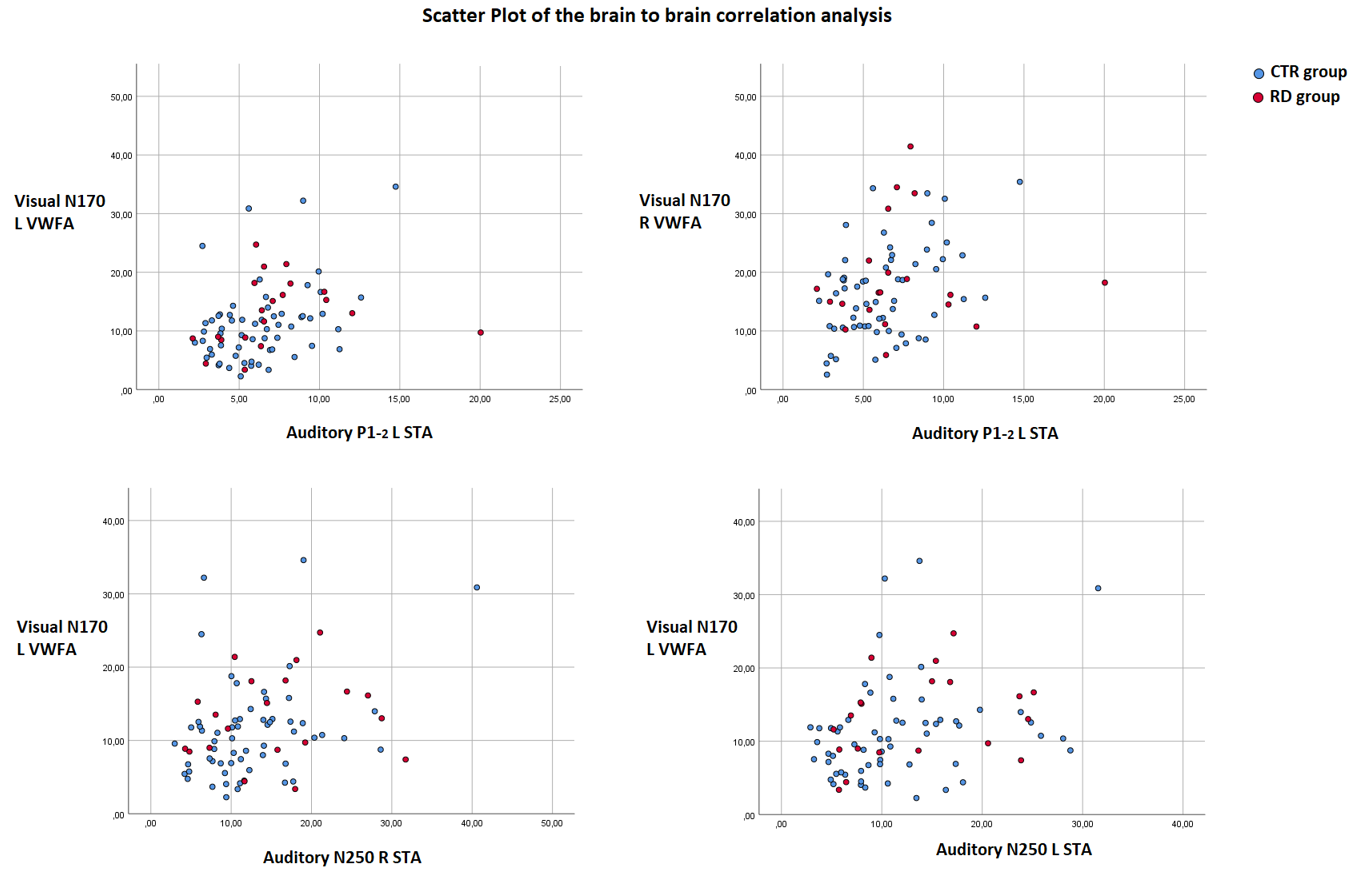


Supplementary Figure 2. Brain-to-brain correlation scatter plots of the significant results (after FDR correction) showing the groups (CTR and RD) distributed within the CTRD continuum

# Supplementary Tables

Supplementary Table 1. Skewness test of the CTRD group

|  |  |  | Statistics | Standard Error |
| --- | --- | --- | --- | --- |
| PAF | Mean |  | -,302655750 | ,1053530419 |
|  | 95%confidence interval for mean | Lower Bound | -,212355734 |  |
|  |  | Upper bound | -,092955766 |  |
|  | 5% Trimmed Mean |  | -,322026806 |  |
|  | Median |  | -,355305000 |  |
|  | Variance |  | ,888 |  |
|  | Std. Deviation |  | ,9423062532 |  |
|  | Minimum |  | -2,4045900 |  |
|  | Maximum |  | 2,7480600 |  |
|  | Range |  | 5,1526500 |  |
|  | Interquartile range |  | 1,3871875 |  |
|  | Skewness |  | ,349 |  |
|  | Kurtosis |  | ,401 |  |

Supplementary Table2. Test of Normality

| Kolmogorov-Smirnov^a^ | | | | Shapiro-Wilk | | |
| --- | --- | --- | --- | --- | --- | --- |
|  | Statistic | df | Sig. | Statistic | df | Sig. |
| PAF | ,055 | 80 | ,200* | ,990 | 80 | ,778 |

Note: *. This a lower bound of the true significance; a. Lilliefors Significance Correction

Supplementary Table3. Correlation results of the brain-to-brain analysis by group and the comparison of the correlation coefficients

|  | Sources | CTR group  N=60 | RD group  N=20 | Correlation comparison | |
| --- | --- | --- | --- | --- | --- |
| Component |  |  |  | Z value  (Fisher 1925) | p-value |
| Auditory P1_2 * Visual N170 | Aud L STA * Vis L VWFA | r= ,387 | r= ,191 | ,7776 | ,4368^n^ |
| Auditory P1_2 * Visual N170 | Aud L STA * Vis R VWFA | r= ,435 | r= ,067 | ,4436 | ,1488^n^ |
| Auditory N250 * Visual N170 | Aud R STA * Vis L VWFA | r= ,329 | r= ,131 | ,7597 | ,4474^n^ |
| Auditory N250 * Visual N170 | Aud L STA * Vis L VWFA | r= ,270 | r= ,292 | - ,0864 | ,9311^n^ |

Note: α=0.05; ^n^ Null hypothesis retained
